# Harvard University, Department of Veterinary Medicine

**Published:** 1891-07

**Authors:** 


*Harvard University, Department of Veterinary Medicine.*—Frederick Huntington Osgood, B.S., M.R.C.V.S., has been appointment instructor in Cattle Practice.

William Orison Underwood, A.B., has been appointed Lecturer in Warranty and Evidence. The Museum and Library has been considerably enlarged.

